# The clinical characteristics of Chinese elderly patients with different durations of type 2 diabetes mellitus

**DOI:** 10.3389/fendo.2022.904347

**Published:** 2022-07-29

**Authors:** Yun Yu, Kaipeng Xie, Qinglin Lou, Hui Xia, Dan Wu, Lingli Dai, Cuining Hu, Shan Shan, Kunlin Wang, Wei Tang

**Affiliations:** ^1^ Department of Endocrinology and Metabolism, Geriatric Hospital of Nanjing Medical University, Nanjing, China; ^2^ Department of Public Health, Nanjing Maternity and Child Health Care Hospital, Women’s Hospital of Nanjing Medical University, Nanjing, China

**Keywords:** clinical characteristics, elderly patients, type 2 diabetes mellitus, duration of diabetes, control targets

## Abstract

**Aims:**

To explore the clinical characteristics among elderly (aged ≥60 years) patients with type 2 diabetes (T2DM) of different durations.

**Methods:**

Clinical characteristics were investigated in 3840 elderly T2DM patients according to their different durations of diabetes (< 1 year, 1~5 years, 5~10 years, and ≥ 10 years). Kruskal-Wallis and Dunn tests were used to assess the differences among groups for continuous variables. The chi-square and *post hoc* tests were carried out for dichotomous variables. The logistic regression was adopted to investigate the relationships between various durations of diabetes and the control rates of achieving the control targets for T2DM as well as diabetic vascular complications.

**Results:**

There were 972, 896, 875 and 1097 patients with a duration of diabetes of <1, 1~5, 5~10 and ≥10 years, respectively. In logistic regression models adjusted for age, sex, education, BMI, smoking and family history of diabetes, elderly T2DM patients with a duration of diabetes of ≥10 years were more likely to reach the comprehensive control targets for TC (OR_TC_ = 1.36, 95% CI =1.14-1.63), LDL-C (OR_LDL-C_ = 1.39, 95% CI =1.17-1.66), TG (OR_TG_ = 1.76, 95% CI =1.46-2.12) and BMI (OR_BMI_ = 1.82, 95% CI =1.52-2.18). Elderly T2DM patients with a duration of diabetes of 1~5 years were more likely to achieve the HbA1c control target (OR_HbA1c_ = 1.92, 95% CI = 1.59-2.31) than elderly T2DM patients with a duration of diabetes of <1 year. Furthermore, in elderly T2DM patients with a duration of diabetes of 5~10 years or ≥ 10 years, the duration of diabetes was positively associated with diabetic macrovascular complications (coronary heart disease and peripheral artery disease). In elderly T2DM patients with a duration of diabetes of over 10 years, the duration of diabetes was associated with diabetes kidney disease (all *P* < 0.05).

**Conclusions:**

It is worth noting that the clinical characteristics of elderly patients with type 2 diabetes in different durations of diabetes are different.

## Introduction

Diabetes is a metabolic disease characterized by chronic hyperglycaemia that is prevalent in China and globally. The global diabetes map (Ninth Edition), released by the International Diabetes Federation, shows that the number of patients with diabetes worldwide is 463 million currently and will increase to 700 million by 2045. China has the largest number of patients with diabetes and the largest elderly diabetic population (1). The ageing population and increasing elderly diabetic population have placed a heavy burden on health systems and the social economy (2, 3).

Type 2 diabetes mellitus (T2DM) is the most common clinical type of diabetes, accounting for nearly 90% of all diabetic cases (4). In addition to the damage resulting from hyperglycaemia and diabetic macrovascular and microvascular complications, the harmful effects of T2DM are mainly caused by comorbidities such as hypertension, dyslipidaemia, overweight and obesity. These conditions could result in a low quality of life and shortened life expectancy (5).

According to the guidelines of prevention and treatment for Chinese T2DM (2020 edition), in addition to achieving glycosylated haemoglobin (HbA1c) < 7% and maintaining glucose levels within the target range, the comprehensive management of T2DM also includes the control of other risk factors, such as hypertension, dyslipidaemia, overweight and obesity (6). Good T2DM comprehensive management can reduce the incidence of diabetic complications and comorbidities (7). Our previous research found that elderly patients (aged ≥ 80 years) with T2DM were more likely to achieve HbA1c and lipid profile targets than younger patients (aged < 60 years) (8). However, the impact of diabetes duration on the comprehensive management of T2DM remains to be explored. Furthermore, since age and diabetic duration are both major risk factors for diabetic complications (9), it is crucial to understand the differences in clinical characteristics in T2DM patients with different durations of diabetes, especially in elderly individuals.

In the present cross-sectional study, we aimed to explore the relationships between diabetes duration and the comprehensive management of T2DM as well as diabetic vascular complications in Chinese elderly patients with T2DM.

## Methods

### Study population

A population of 5516 elderly patients with T2DM (aged ≥ 60 years) visited the Geriatric Hospital of Nanjing Medical University in Nanjing, China, between Jan 2013 and Dec 2020. The diagnosis of T2DM was based on the diagnostic criteria of the World Health Organization in 1999 (10). After excluding those who had a history of type 1 diabetes, latent autoimmune diabetes in adults (LADA), or secondary diabetes or those who had any missing data for key variables including blood pressure, HbA1c, total cholesterol (TC), low density lipoprotein cholesterol (LDL-C), high-density lipoprotein cholesterol (HDL-C) triglyceride (TG) and body mass index (BMI), a total of 3840 elderly patients with T2DM were finally eligible for analysis. Patients were divided into four groups according to diabetic duration: < 1 year (Group 1), 1~5 years (Group 2), 5~10 years (Group 3), and ≥ 10 years (Group 4). The Ethics Committee of the Geriatric Hospital of Nanjing Medical University approved the study protocol. Each patient signed written informed consent.

### Data sources

The clinical data of all elderly patients with T2DM were collected from the electronic medical record system. These information included diabetes duration, age, sex, education level, smoking status, family history of diabetes, systolic pressure (SBP), diastolic pressure (DBP), BMI, HbA1c, TG, TC, LDLC, HDL-C, therapeutic regimens, such as diabetes treatment (diet and exercise alone and anti-hyperglycaemic agents), and antihypertensive agents. Education level was defined as ≥ 9 years or < 9 years. Smoking status was divided into two groups, current smoking and non-current smoking. BMI was calculated as weight (kg)/height (m)^2^. Overnight fasting venous blood samples were obtained to measure HbA1c. All blood samples were measured at the laboratory of the Geriatric Hospital of Nanjing Medical University.

The data of macrovascular and microvascular complications of diabetes were also collected based on the available information. The diagnosis of coronary heart disease (CHD) was based on a history of angina pectoris, myocardial infarction, percutaneous coronary intervention or coronary artery bypass. The diagnosis of peripheral artery disease (PAD) was based on an ankle-brachial pressure index (ABI) < 0.9. The estimated glomerular filtration rate (eGFR) was calculated by the CKD-EPI formula (11), and the diagnosis of diabetes kidney disease (DKD) was based on the KDIGO clinical practice guidelines (12, 13). Albuminuria was defined as a urine albumin/creatinine ratio (ACR) > 30 mg/gCr. Patients with eGFR < 60 ml·min^-1^ ·1.73m^2^ or albuminuria were defined as having DKD.

### Comprehensive control targets for T2DM

According to the guidelines for Chinese T2DM (2020 edition) (6), the T2DM control targets of comprehensive management include BP<130/80 mmHg, HbA1c < 7%, TC < 4. 5 mmol/L, LDL-C < 2. 6 mmol/L, HDL-C >1.0 mmol/L (males) or >1.3 mmol/L(females), TG< 1. 7 mmol/L, and BMI < 24.0 kg/m^2^.

### Statistical analysis

Our clinical characteristics were non-normally distributed, descriptive information was presented as median (interquartile range [IQR]) for continuous variables or numbers and percentages for categorical variables. For continuous variables, Kruskal-Wallis and *post hoc* multiple comparison Dunn’s tests were used to test for the differences (R package FSA). For categorical variables, χ2 tests and *post hoc* tests were carried out to examine the significant differences (R package companion). Logistic regression analysis was used to calculate the odds ratios (ORs) and 95% confidence intervals (95% CIs) for the T2DM achieved control targets or the associations of different diabetic duration groups with the risks of diabetic macrovascular and microvascular complications. The unadjusted ORs and 95% CIs were calculated in Model 1. Two other models were progressively adjusted for age, sex, education, BMI (Model 2), smoking and family history of diabetes (Model 3). Patients with a diabetes duration < 1 year were the reference group. *P* values for trend were calculated by modeling ordinal categories as continuous variables. A two-tailed threshold of *P* < 0.05 was considered statistically significant. Missing data was presented as not available and were not taken into consideration during analysis. The statistics were calculated using R statistical software version 4.1.0.

## Results

### Baseline characteristics of elderly patients with T2DM among different diabetic duration groups

The characteristics of elderly patients with T2DM according to different diabetic duration groups are described in ###$###

**Table 1**. A total of 3840 elderly patients with T2DM were classified as group 1 (n = 972), group 2 (n = 896), group 3 (n = 875) and group 4 (n = 1097). Between the four groups, there were no significant differences in age, SBP, the proportion of higher education, current smoking and patients who used antihypertensive agents (all *P* > 0.05). In group 4, compared with groups 1 and 2, there was a significantly decreased percentage of male (*P*_group 4_ *_vs_*_group 1_ = 0.013, *P*_group 4_ *_vs_*_group 2 =_ 0.035) and lower levels of DBP (*P*_group 4_ *_vs_*_group 1_ < 0.001, *P*_group 4_ *_vs_*_group 2_ = 0.014), BMI (*P*_group 4_ *_vs_*_group 1_ <0.001, *P*_group 4_ *_vs_*_group 2_ < 0.001), TG (*P*_group 4_ *_vs_*_group 1_ < 0.001, *P*_group 4_ *_vs_*_group 2_ < 0.001) and TC (*P*_group 4_ *_vs_*_group 1_ < 0.001, *P*_group 4_ *_vs_*_group 2_ = 0.018), whereas the increased percentage of family history of diabetes (*P*_group 4_ *_vs_*_group 1_ < 0.001, *P*_group 4_ *_vs_*_group 2_ < 0.001). With prolonged diabetic status, there was a significantly decreasing trend in the proportion of patients who used diet and exercise alone to control blood glucose (28.70%, 16.96%, 10.06%, 3.92%, *P* < 0.001). Compared to group 1, the level of HbA1c was significantly higher in group 4 (7.40% *vs* 7.10%, *P* = 0.015), but was significantly lower in group 2 (6.70% *vs* 7.10%, *P* < 0.001) and group 3 (7.00% *vs* 7.10%, *P* = 0.002).

**Table 1:** Characteristics of elderly T2DM patients with different diabetic durations.

Characteristics		Diabetic duration				*P* value
		Group 1 ( < 1 year)	Group 2 (1-5 years)	Group 3 (5-10 years)	Group 4 (≥10 years)	
Number of patients		972	896	875	1097	
Age (years)		70 (65 - 77)	70 (64 - 76)	71 (65 - 77)	70 (65 - 76)	0.297
Sex						0.002
	Male	602 (61.93)^a^	550 (61.38)^a^	489 (55.89) ^a,b^	605 (55.15) ^b,c^	
	Female	370 (38.07)	346 (38.62)	386 (44.11)	492 (44.85)	
Education [n (%)]†						0.570
	≥9 years	658 (67.70)	591 (65.96)	567 (64.80)	719 (65.54)	
	<9 years	313 (32.20)	305 (34.04)	308 (35.20)	378 (34.46)	
Current smoking [n (%)]						0.758
	Yes	188 (19.34)	168 (18.75)	157 (17.94)	217 (19.78)	
	No	784 (80.66)	728 (81.25)	718 (82.06)	880 (80.22)	
Family history of diabetes [n (%)]						< 0.001
	Yes	298 (30.66)^a^	296 (33.04)^a,b^	332 (37.94)^b,c^	516 (47.04)^d^	
	No	674 (69.34)	600 (66.96)	543 (62.06)	581 (52.96)	
SBP (mmHg)		130 (120 - 140)^a^	130 (120 - 140)^a^	130 (120 - 140)^a^	130 (120 - 140)^a^	0.045
DBP (mmHg)		80 (70 - 80)^a^	80 (70 - 80)^a^	78 (70 - 80)^a,b^	77 (70 - 80)^b^	< 0.001
BMI (kg/m^2^)		25.2 (23.4 - 27.4)^a^	25.1 (23.0 - 27.3)^a^	25.0 (22.9 - 27.0)^a^	24.2 (22.3 - 26.5)^b^	< 0.001
HbA1c (%)		7.10 (6.40 - 9.13)^a^	6.70 (6.20 - 7.60)^b^	7.00 (6.40 - 8.00) ^c^	7.40 (6.60 - 9.00)^d^	< 0.001
TG (mmol/L)		1.50 (1.09 - 2.03)^a^	1.40 (1.00 - 1.96)^b^	1.31 (0.95 - 1.90)^b^	1.26 (0.91 - 1.76)^c^	< 0.001
TC (mmol/L)		4.74 (4.10 - 5.44)^a^	4.71 (3.99 - 5.40) ^a^	4.66 (3.96 - 5.35) ^a,b^	4.54 (3.93 - 5.23) ^b,c^	< 0.001
LDL - C (mmol/L)		2.81 (2.21 - 3.42)^a^	2.74 (2.16 - 3.28)^a,b^	2.70 (2.19 - 3.30)^a,b^	2.63 (2.10 - 3.20) ^b^	< 0.001
HDL - C (mmol/L)		1.12 (0.96 - 1.35)^a^	1.15 (0.99 - 1.39)^a^	1.17 (0.98 - 1.39)^a^	1.17 (0.97 - 1.40)^a^	0.019
Diabetes treatment [n (%)]						
	Diet and exercise alone	279 (28.70)^a^	152 (16.96)^b^	88 (10.06)^c^	43 (3.92)^d^	< 0.001
	Others*	693 (71.30)	744 (83.04)	787 (89.94)	1054 (96.08)	
Antihypertensive agents [n (%)]						0.068
	Yes	538 (55.35)	544 (60.71)	492 (56.23)	648 (59.07)	
	No	434 (44.65)	352 (39.29)	383 (43.77)	449 (40.93)	

Data are expressed as median (Q1-Q3) or numbers and percentages, n (%); P values for comparison over all 4 categories.

^a,b,c,d^ Groups with the same superscript letters are not significantly different.

^†^Education information is not available in one patient.

*Others include anti-hyperglycemic agents, such as insulin and oral antidiabetic drugs.

### Control rates of T2DM targets among elderly patients with different durations

The control rates of T2DM targets in elderly patients with T2DM between different groups are shown in **Table 2**. Compared to group 1, the control rate of HbA1c was higher in group 2 (61.61% vs 45.86%, *P* < 0.001), whereas the rate was significantly lower in group 4 (36.55% *vs* 45.86%, *P* < 0.001). Group 4 had a significantly higher control rate of TC when compared with groups 1 (47.77% *vs* 41.15%, *P* = 0.018). The similar results were observed for the control rates of LDL-C (47.86% *vs* 39.92%, *P* = 0.002). Patients of group 4 were more likely to be higher control rate of TG and BMI when compared with other groups (all *P* < 0.05). Additionally, no significant differences in the control rates of blood pressure and HDL-C were found across the groups with various durations of diabetes (all *P* > 0.05).

**Table 2:** Control rates of elderly T2DM patients with different diabetic durations.

Characteristics		Diabetic duration				*P* value
		Group 1 (< 1 year)	Group 2 (1-5 years)	Group 3 (5-10 years)	Group 4 ( ≥ 10 years)	
Number of patients		972	896	875	1097	
Blood pressure (mmHg)						0.333
	SBP<130 and DBP<80	266 (27.37)	258 (28.79)	273 (31.20)	324 (29.54)	
	≥ 130/80	706 (72.63)	638 (71.21)	602 (68.80)	773 (70.46)	
HbA1c (%)						< 0.001
	< 7	444 (45.68)^a^	552 (61.61)^b^	433 (49.49)^a^	401 (36.55)^c^	
	≥ 7	528 (54.32)	344 (38.39)	442 (50.51)	696 (63.45)	
TC (mmol/L)						0.010
	< 4.5	400 (41.15)^a^	374 (41.74)^a,b^	384 (43.89)^a,b^	524 (47.77)^b,c^	
	≥ 4.5	572 (58.85)	522 (58.26)	491 (56.11)	573 (52.23)	
LDL-C (mmol/L)						0.003
	< 2.6	388 (39.92)^a^	394 (43.97)^a,b^	400 (45.71)^a,b^	525 (47.86)^b,c^	
	≥ 2.6	584 (60.08)	502 (56.03)	475 (54.29)	572 (52.14)	
HDL-C (mmol/L)						0.083
	Achieved target*	513 (52.78)	523 (58.37)	487 (55.66)	592 (53.97)	
	Did not achieve target*	459 (47.22)	373 (41.63)	388 (44.34)	505 (46.03)	
TG (mmol/L)						< 0.001
	< 1.7	580 (59.67)^a^	577(64.40)^a,b^	587 (67.09)^b^	800 (72.93)^c^	
	≥ 1.7	392 (40.33)	319(35.60)	288 (32.91)	297 (27.07)	
BMI (kg/m^2^)						< 0.001
	< 24	310 (31.89)^a^	313 (34.93)^a^	314 (35.89)^a,b^	503 (45.85)^c^	
	≥ 24	662 (68.11)	583 (65.07)	561 (64.11)	594 (54.15)	

Data are expressed as numbers and percentages, n (%); P values for comparison over all 4 categories.

^a,b,c^ Groups with the same superscript letters are not significantly different. *Achieved target means the levels of HDL-C >1.0 mmol/L in males or >1.3 mmol/L in females; Did not achieve target means the levels of HDL-C≤1.0 mmol/L in males or ≤1.3 mmol/L in females.

As shown in **Table 3**, after adjusting for age, sex, education, BMI, smoking and family history of diabetes (model 3), T2DM patients in group 2 had significantly increased OR for achieving the control target for HbA1c (OR_HbA1c_ = 1.92, 95% CI = 1.59-2.31, *P* < 0.001), while elderly T2DM patients in group 4 showed significantly decreased OR for achieving the control target for HbA1c (OR_HbA1c_ = 0.65, 95% CI = 0.54-0.78, *P* < 0.001). The significantly increased ORs for achieving the control targets for TC (OR_TC_ = 1.36, 95% CI =1.14-1.63, *P* < 0.001), LDL-C (OR_LDL-C_ = 1.39, 95% CI =1.17-1.66, *P* < 0.001) and BMI (OR_BMI_ = 1.82, 95% CI =1.52-2.18, *P* < 0.001) were observed in group 4. Interestingly, we observed the gradually increased ORs for achieving the control targets for TC with the prolonged duration of diabetes (OR_group 2_ = 1.75, 95% CI =1.45-2.12; OR_group 3_ = 1.38, 95% CI =1.13-1.68; OR_group 4_ = 1.76, 95% CI =1.46-2.14; *P* < 0.001) in model 3.

**Table 3:** Odds ratios (95% CI) for achieved comprehensive control targets by different diabetic duration groups among elderly patients with T2DM (Ref. < 1 year).

Characteristics		Diabetic duration			*P*_trend_
		Group 2 (1-5 years)	Group 3 (5-10 years)	Group 4 (≥ 10 years)	
HbA1c < 7%					
	Model 1	1.91 (1.59,2.29)	1.16 (0.97,1.40)	0.69 (0.57,0.82)	< 0.001
	Model 2	1.91 (1.59,2.30)	1.14 (0.94,1.37)	0.64 (0.53,0.76)	< 0.001
	Model 3	1.92 (1.59,2.31)	1.15 (0.95,1.38)	0.65 (0.54,0.78)	<0.001
BP < 130/80 mmHg					
	Model 1	1.07 (0.88,1.31)	1.20 (0.98,1.47)	1.11 (0.92,1.35)	0.187
	Model 2	1.06 (0.86,1.3)	1.15 (0.94,1.42)	1.00 (0.82,1.21)	0.921
	Model 3	1.06 (0.86,1.3)	1.16 (0.94,1.42)	1.00 (0.82,1.22)	0.844
TC < 4.5 mmol/L					
	Model 1	1.02 (0.85,1.23)	1.12 (0.93,1.35)	1.31 (1.10,1.56)	0.001
	Model 2	1.03 (0.86,1.24)	1.15 (0.96,1.39)	1.37 (1.15,1.64)	< 0.001
	Model 3	1.03 (0.86,1.24)	1.15 (0.95,1.39)	1.36 (1.14,1.63)	< 0.001
LDL-C < 2.6 mmol/L					
	Model 1	1.18 (0.98,1.42)	1.27 (1.05,1.52)	1.38 (1.16,1.65)	< 0.001
	Model 2	1.18 (0.98,1.42)	1.27 (1.05,1.53)	1.39 (1.16,1.65)	< 0.001
	Model 3	1.18 (0.98,1.42)	1.27 (1.06,1.53)	1.39 (1.17,1.66)	< 0.001
HDL-C Achieved target*					
	Model 1	1.25 (1.04,1.51)	1.12 (0.93,1.35)	1.05 (0.88,1.25)	0.932
	Model 2	1.26 (1.04,1.52)	1.17 (0.97,1.42)	1.04 (0.87,1.24)	0.928
	Model 3	1.25 (1.04,1.52)	1.18 (0.97,1.43)	1.06 (0.88,1.27)	0.742
TG < 1.7 mmol/L					
	Model 1	1.22 (1.01,1.47)	1.38 (1.14,1.67)	1.82 (1.51,2.19)	< 0.001
	Model 2	1.22 (1.01,1.48)	1.37 (1.13,1.67)	1.75 (1.45,2.11)	< 0.001
	Model 3	1.22 (1.01,1.48)	1.38 (1.13,1.68)	1.76 (1.46,2.14)	< 0.001
BMI < 24 (kg/m^2^)^†^					
	Model 1	1.15 (0.95,1.39)	1.20 (0.99,1.45)	1.81 (1.51,2.16)	< 0.001
	Model 2	1.15 (0.95,1.40)	1.19 (0.98,1.44)	1.80 (1.51,2.16)	< 0.001
	Model 3	1.16 (0.95,1.40)	1.19 (0.98,1.45)	1.82 (1.52,2.18)	< 0.001

Model 1: unadjusted model.

Model 2: adjusted for age, sex, education and BMI.

Model 3: adjusted for age, sex, education and BMI, smoking and family history of diabetes.

*Achieved target means the levels of HDL-C >1.0 mmol/L in males or >1.3 mmol/L in females.

^†^Adjusted for age, sex and education in mode 2, and adjusted for age, sex, education, smoking and family history of diabetes in model 3.

### Macrovascular and microvascular complications of diabetes among the groups

The prevalence of diabetic macrovascular and microvascular complications among the groups are presented in **Table 4**. Notably, there are 2157 (56.18%) patients with missing PAD data, 1214 (31.61%) with missing albuminuria, and 114 (2.97%) with missing eGFR. Between the four groups, there was no significant difference in the percentage of patients with eGFR<60 ml·min-1 ·1.73m^2^ (*P* = 0.420). Compared to group1, group 4 had the higher percentage of patients with CHD, PAD and albuminuria (all *P* < 0.05). As shown in **Table 5**, after adjusting for potential confounders, we observed elderly T2DM patients in groups 3 and 4 had significantly increased ORs for CHD (OR_group 3 =_ 1.43, 95% CI =1.07-1.89; OR_group 4 =_ 1.56, 95% CI =1.19-2.04; *P*_trend_< 0.001) and for PAD (OR_group 3 =_ 1.82, 95% CI =1.10-3.01; OR_group 4 =_ 1.60, 95% CI =1.00-2.55; *P*_trend_= 0.022) in model 3. Patients in group 4 had significantly increased ORs for albuminuria (OR _albuminuria_ = 1.96, 95% CI =1.55-2.48, *P*_trend_ < 0.001; OR _eGFR_ = 1.36, 95% CI =1.05-1.75, *P*_trend_ =0.030).

**Table 4:** Characteristics of elderly T2DM patients with diabetic complications.

Characteristics		n (%)	Diabetic duration				*P* value
			Group 1 (< 1 year)	Group 2 (1-5 years)	Group 3 (5-10 years)	Group 4 (≥10 years)	
Total number of patients		3840 (100)	972	896	875	1097	
Macrovascular complications							
CHD							
	Yes	504 (13.13)	101 (10.39)^a^	102 (11.38)^a,b^	128 (14.63)^b,c^	173 (15.77)^c^	0.001
	No	3336 (86.87)	871 (89.61)	794 (88.62)	747 (85.37)	924 (84.23)	
PAD^*^							
	Yes	181 (4.71)	29 (2.98)^a^	25 (2.79)^a,b^	47 (5.37)^b^	80 (7.29)^b^	0.001
	No	1502 (39.11)	398 (40.95)	284 (31.70)	313 (85.37)	507 (46.22)	
Microvascular complications							
DKD (Albuminuria)^†^							
	Yes	786 (20.47)	150 (15.43)^a^	148 (16.52)^a^	173 (19.77)^a^	315 (28.71)^b^	< 0.001
	No	1840 (47.92)	501 (51.54)	403 (44.98)	426 (48.69)	510 (46.49)	
DKD (eGFR<60 ml·min^-1^ ·1.73m^2^)^‡^							0.420
	Yes	668 (17.40)	156 (16.05)	153 (17.08)	154 (17.60)	205 (18.69)	
	No	3058 (79.64)	794 (81.69)	711 (79.35)	694 (79.31)	859 (78.30)	

CHD, Coronary heart disease. PAD, Peripheral vascular disease. DKD, diabetes kidney disease.

Data are expressed as numbers and percentages, n (%); P values for comparison over all 4 categories.

^*^PAD information is available in 1683 (43.83%) patients.

^†^Albuminuria (ACR > 30 mg/gCr). ACR information is available in 2626 (68.39%) patients.

^‡^eGFR is available in 3726 (97.03%) patients.

^a,b,c^Groups with the same superscript letters are not significantly different.

**Table 5:** Odds ratios (95% CI) for macrovascular and microvascular complications by different diabetic duration groups among elderly patients with T2DM (Ref. < 1 year).

Characteristics		Diabetic duration			*P*_trend_
		Group 2 (1-5 years)	Group 3 (5-10 years)	Group 4 ( ≥ 10 years)	
Macrovascular complications					
CHD					
	Model 1	1.11 (0.83,1.48)	1.48 (1.12,1.95)	1.61 (1.24,2.10)	< 0.001
	Model 2	1.13 (0.84,1.51)	1.44 (1.09,1.91)	1.60 (1.23,2.09)	< 0.001
	Model 3	1.12 (0.84,1.51)	1.43 (1.07,1.89)	1.56 (1.19,2.04)	< 0.001
PAD					
	Model 1	1.21 (0.69,2.11)	2.06 (1.27,3.35)	2.17 (1.39,3.38)	< 0.001
	Model 2	1.12 (0.63,1.98)	1.83 (1.11,3.03)	1.64 (1.03,2.60)	0.016
	Model 3	1.11 (0.63,1.97)	1.82 (1.10,3.01)	1.60 (1.00,2.55)	0.022
Microvascular complications					
DKD (Albuminuria)					
	Model 1	1.23 (0.94,1.59)	1.36 (1.05,1.75)	2.06 (1.64,2.6)	< 0.001
	Model 2	1.21 (0.93,1.57)	1.29 (1.00,1.67)	1.96 (1.55,2.47)	< 0.001
	Model 3	1.21 (0.93,1.58)	1.29 (1.00,1.67)	1.96 (1.55,2.48)	< 0.001
DKD (eGFR < 60 ml·min^-1^ ·1.73m^2^)					
	Model 1	1.10 (0.86,1.40)	1.13 (0.88,1.44)	1.21 (0.97,1.53)	0.097
	Model 2	1.16 (0.88,1.51)	1.11 (0.85,1.46)	1.32 (1.03,1.71)	0.047
	Model 3	1.16 (0.89,1.52)	1.12 (0.86,1.47)	1.36 (1.05,1.75)	0.030

Model 1: unadjusted model.

Model 2: adjusted for age, sex, education and BMI.

Model 3: adjusted for age, sex, education, BMI, smoking and family history of diabetes.

## Discussion

In the current study, we evaluated the comprehensive control of T2DM and diabetic complications in elderly patients stratified by different durations of diabetes. Elderly T2DM patients with a duration of diabetes of ≥ 10 years were more likely to achieve the comprehensive control targets for TC, LDL-C and TG, while elderly T2DM patients with a duration of diabetes of 1~5 years were more likely to achieve the HbA1c control target than elderly T2DM patients with a duration of diabetes of < 1 year. In elderly T2DM patients with a duration of diabetes of 5~10 years or ≥ 10 years, the duration of diabetes was independently associated with diabetic macrovascular complications (CHD and PAD). In addition, in patients with a duration of diabetes of ≥ 10 years, the duration of diabetes was independently associated with the risk of DKD.

HbA1c is typically used as the gold standard for evaluating glycaemic control and is a clinical indicator for predicting diabetic complications (4). In addition, HbA1c < 7% is the standard for good glycaemic control in most adults with T2DM that is recommended by most guidelines (4, 6). Previous studies have shown that the ORs of poor glycaemic control increase with diabetes duration in T2DM (14, 15). Interestingly, in the current study, elderly T2DM patients with a duration of diabetes of 1~5 years were more likely to achieve the HbA1c control target than those who had a duration of diabetes less than 1 year, and the control target rate of HbA1c gradually decreased with the extension of the duration. The reasons for this finding are still unclear but may be caused by differences in study design, the deterioration of islet function with diabetes progression (16), or the clinical criteria for glycaemic control (17).

Dyslipidaemia is one of the most important risk factors for CHD, and optimal lipid control can improve cardiovascular outcomes (18). Our study found that the higher control rates for TC, LDL-C, TG and BMI were observed in elderly T2DM patients with a duration of diabetes of ≥ 10 years than that in patients who had a duration of diabetes less than 1 year. A possible reason is that since the similar prevalence of CHD and PAD was observed in patients with a duration of diabetes of ≥ 10 years, elderly patients with T2DM may pay more prone to it. Another reason is that the elderly patients with long duration may use lipid-lowering or antiplatelet agents to relieve symptoms. Further studies with more detail information should be performed to explore the underlying reasons.

Previous cohort studies have reported that the duration of diabetes is associated with the risk of developing diabetes-related complications in aged T2DM patients (19, 20). One study in the USA showed that the prevalence of CHD, PAD and cerebrovascular disease was significantly higher in elderly T2DM patients (aged ≥ 60 years) with a duration of diabetes ≥ 10 years than in those with a shorter duration (19). Furthermore, another study in Australia reported that the incidences of myocardial infarction and stroke-related death increased along with an increase in the duration of diabetes in 1433 aged male patients with diabetes (aged ≥ 65 years) (20). Our study also found that elderly T2DM patients with a duration of diabetes of 5~10 years or ≥ 10 years were more likely to develop diabetic macrovascular complications (CHD and PAD) than those with a duration of diabetes of <1 year, which was consistent with the abovementioned studies. Moreover, the present study also suggested that the duration of diabetes was significantly associated with microvascular complications (albuminuria), which is in accordance with a previous study (21).

There were several limitations in our study. First, there is missing data on outcomes of interest including PAD and DKD, and our study was a cross-sectional and single-center study. Future large and longitudinal studies with these outcomes are needed to confirm the associations. Second, the preference of doctors on medication regiment and drug choice might influence the analytic data on characteristics of the elderly T2DM patients based on diabetes duration. Data on the detail medication and medication adherence of patients should be considered into further studies.

In summary, elderly T2DM patients with a duration of diabetes of 1~5 years were more likely to achieve the HbA1c control target, while patients with a duration of diabetes of ≥ 10 years were more likely to achieve the comprehensive control targets for the lipid profile. In addition, the duration of diabetes was independently associated with diabetic macrovascular complications (CHD and PAD) in elderly T2DM patients with a duration of diabetes of 5~10 years or ≥ 10 years and was significantly associated with DKD in patients a duration of diabetes of with ≥ 10 years.

## Data availability statement

The raw data supporting the conclusions of this article will be made available by the corresponding author, without undue reservation.

## Author contributions

WT and YY conceived and design the research. YY and KX analyzed the data. QL, HX, DW, LD, CH, SS, and KW collected the data. YY and KX wrote and revised the initial manuscript. WT supervised the whole study and revised the manuscript. All authors contributed to the article and approved the submitted version.

## Funding

This study was supported by grants from the National Natural Science Foundation of China (81770773), Natural Science Foundation of Jiangsu Province (BK20211375), the Foundation of Jiangsu Province Health Bureau (BJ18008), and Cohort study of elderly type 2 diabetes from Nanjing Medical University (NMUC2020041).

## Conflict of interest

The authors declare that the research was conducted in the absence of any commercial or financial relationships that could be construed as a potential conflict of interest.

## Publisher’s note

All claims expressed in this article are solely those of the authors and do not necessarily represent those of their affiliated organizations, or those of the publisher, the editors and the reviewers. Any product that may be evaluated in this article, or claim that may be made by its manufacturer, is not guaranteed or endorsed by the publisher.
